# Construction and application of procedural pathways combined with information management in nursing staff skills training system

**DOI:** 10.1186/s12909-024-05593-x

**Published:** 2024-06-21

**Authors:** Liping Cui, Yuting Dong, Shan Zhang, Wenxia Ma, Min Li

**Affiliations:** 1 Department of nursing, Tongji Shanxi Hospital, Shanxi Bethune Hospital, Shanxi Academy of Medical Sciences, Third Hospital of Shanxi Medical University, Taiyuan, 030032 China; 2 Department of ICU, Tongji Shanxi Hospital, Shanxi Bethune Hospital, Shanxi Academy of Medical Sciences, Third Hospital of Shanxi Medical University, Taiyuan, 030032 China; 3 Department of general surgery, Tongji Shanxi Hospital, Shanxi Bethune Hospital, Shanxi Academy of Medical Sciences, Third Hospital of Shanxi Medical University, Taiyuan, 030032 China; 4 Department of Breast Surgery, Tongji Shanxi Hospital, Shanxi Bethune Hospital, Shanxi Academy of Medical Sciences, Third Hospital of Shanxi Medical University, Taiyuan, 030032 China; 5 Tongji Hospital, Tongji Medical College, Huazhong University of Science and Technology, Wuhan, 430030 China; 6 Department of Breast Surgery, Tongji Shanxi Hospital, Tongji Hospital, Tongji Medical College, Shanxi Bethune Hospital, Shanxi Academy of Medical Sciences, Third Hospital of Shanxi Medical University, Huazhong University of Science and Technology, No. 99 of Longcheng Street, Xiaodian District, Taiyuan, 030032 China

**Keywords:** Proceduralization, Informatization, Nursing staff, Skills training system

## Abstract

**Objective:**

To explore the application effect of procedural pathways combined with information management in the construction of nursing staff skills training system.

**Methods:**

This was a quasi-experimental study with a control group and an experimental group. A total of 300 newly admitted nurses or nurses who required training within three years of admission were selected as the experimental group, and 267 nurses who were trained in the same hospital during the same period in 2020 were selected as the control group. The experimental group received skills training using a system that combines procedural pathways with information management, while the control group received traditional teaching mode. The outcome measures included theoretical score, operation score, nurse competency, patient satisfaction, and nursing-related adverse events. The data were analyzed using t-test, chi-square test, and rank-sum test.

**Results:**

The experimental group had higher scores in theoretical assessment, skills assessment, nurse competency, and patient satisfaction, and lower incidence of nursing-related adverse events than the control group (*P* < 0.05).

**Conclusion:**

The strategy of procedural pathways combined with information management provides a new perspective and method for nursing operation skills training, effectively improves clinical nursing quality and ensures patient safety.

## Introduction

Proceduralization refers to describing a certain job in a unified format based on the operation purposes, steps, and requirements, in order to guide and standardize daily work [1]. The National Nursing Career Development Plan (2016–2020) proposes vigorously promoting nursing information construction by taking advantage of the rapid development of information technologies such as big data, cloud computing, the Internet of Things, and mobile communication [2, 3]. Driven by China’s policies and technological development, the informational application of nursing management has achieved remarkable results, effectively improving the quality of nursing services in the country [3]. Nursing skills are essential professional skills for clinical nurses in their clinical work, and the technical level of nursing staff directly affects the quality of clinical nursing [4]. Research [1] has shown that there remain defects in current clinical nursing training, such as unclear objectives, and weak pertinence of training content and form. The training program needs to be further optimized to improve the quality and effect of training. By combining procedural pathways with information management to construct a nursing skills training system, this is expected to cultivate nurses with solid theoretical foundation, proficient operating skills, and clinical problem-solving abilities, promote smooth clinical work, ensure patient safety, and provide a reference for the establishment of a nursing training system.Previous studies have explored various strategies to improve the quality and effectiveness of nursing skills training, such as simulation-based learning, problem-based learning, blended learning, and competency-based training [5]. However, few studies have examined the application effect of procedural pathways combined with information management in the construction of nursing staff skills training system. Procedural pathways are standardized and evidence-based guidelines that describe the operation purposes, steps, and requirements for a certain job, aiming to guide and regulate daily work [6]. Information management is the use of information technologies, such as big data, cloud computing, the Internet of Things, and mobile communication, to collect, store, process, and analyze data related to nursing management and practice, aiming to improve the quality and efficiency of nursing services [7]. These two strategies have been shown to have positive impacts on clinical nursing quality and patient safety individually [8], However, few studies have explored the application effect of procedural pathways combined with information management in the construction of nursing staff skills training system. Therefore, this study aimed to explore the application effect of procedural pathways combined with information management in the construction of nursing staff skills training system.

## Objects and methods

### Research objects

This was a quasi-experimental study with a control group and an experimental group.A total of 300 newly admitted nurses at XX Hospital who required training from January 1, 2021 to December 31, 2021, or nurses who were still in need of training within three years of admission were selected as the experimental group. A total of 267 nurses who were trained in the same hospital during the same period in 2020 were selected as the control group. The training need of nurses was identified based on the following criteria: ①Newly admitted nurses; ②Nurses who required continued training within three years of admission; ③Informed consent and willingness to participate in this study.The nurses who met these criteria were screened from the hospital’s human resources database and invited to join the training program. Exclusion criteria: ①Nurses who withdrew from training midway. Both groups of nurses completed daily nursing tasks in the hospital, and there were no statistically significant differences in their gender, age, education, etc. (*P* > 0.05), ensuring comparability. The two groups had the same internship goals, both based on the 6th edition of “Basic Nursing” and the standardized training syllabus for nurses, combined with the actual clinical needs of the hospital, including patient admission and discharge care, hospital infection prevention and control, patient hygiene and safety care, patient balanced diet care, respiratory care, gastrointestinal care, specimen collection techniques, etc. Through a combination of theory and practice, it enabled nurses to master various clinical nursing skills, cultivated and improved their clinical thinking ability, job competency, communication ability, etc., and promoted them to apply their knowledge to clinical work.The training program was conducted as an in-service training for the nurses, lasting for one year, with monthly sessions of theoretical and skills training.The training syllabus was based on the 6th edition of Basic Nursing and the standardized training syllabus for nurses, combined with the actual clinical needs of the hospital. The training content included patient admission and discharge care, hospital infection prevention and control, patient hygiene and safety care, patient balanced diet care, respiratory care, gastrointestinal care, specimen collection techniques, etc.The control group consisted of 267 nurses who were trained in the same hospital during the same period in 2020. They were selected based on the same criteria as the experimental group, except for the year of admission. They were matched with the experimental group in terms of gender, age, education, and clinical department.Table 1 shows the demographic characteristics of the demographic characteristics of the experimental group and the control group. There were no significant differences between the two groups in terms of gender, age, education, and clinical department (*P* > 0.05), ensuring comparability.

**Table 1:** Demographic characteristics of the experimental group and the control group

Indicator	Experimental Group (*n* = 300)	Control Group (*n* = 267)
Gender (n, %)		
Male	98 (32.67%)	95 (35.58%)
Female	202 (67.33%)	172 (64.42%)
Age (years)	25.6 ± 3.2	25.4 ± 3.1
Education		
Junior college	132 (44%)	125 (46.82%)
Bachelor	168 (56%)	142 (53.18%)
Clinical department		
Internal Medicine	75 (25%)	72 (26.97%)
Surgery	75 (25%)	72 (26.97%)
Pediatrics	50 (16.67%)	48 (17.98%)
Obstetrics and Gynecology	50 (16.67%)	48 (17.98%)

### Research methods

The control group adopted the traditional teaching mode, in which clinical training teachers developed nurse training plans based on content in the training syllabus. The one-on-one teaching mode was used to jointly perform the internship tasks with the trainee nurses, i.e., the traditional nursing internship mode. The training lasted for 1 year, during which lectures on the departmental environment, relevant rules and regulations, precautions, knowledge and skills of common diseases in critical care medicine were given. Basic nursing operation skills, tracheal intubation coordination skills, common critical medical instrument use skills, etc. were taught and checked the next day to assess the trainees’ mastery.

Based on the control group, the experimental group implemented a clinical nursing teaching mode where a training system was constructed by combining procedural pathways with information management. The details are as follows:

#### Faculty construction and training

By organizing multimedia teaching competitions and clinical skills operation assessments in the hospital, 15 nursing staff with 10 years or above continuous clinical nursing work experience from different wards were selected to form a nursing skills operation group. College teachers and clinical nursing experts were invited to teach group members, followed by a one-month standardized and systematic training of skills operation and related knowledge. Based on the characteristics of specialty diseases in the group members’ wards and daily nursing operations, the operation items were divided.

#### Theoretical training

The lecturer of each operation item taught relevant theoretical knowledge and conducted operation demonstrations. The specific procedure is as follows: ①Lecturer lesson preparation: Focusing on clinical problems, the lecturer condensed the teaching content by referring to textbooks, guidelines, consensus, and literature, and formed a lecture outline. ②Online discussion: Two days before the collective lesson preparation, the lecturer sent the lecture outline to the WeChat group of the skills operation group. The group members searched for and proposed questions before the lesson preparation, and solved the problems through evidence-based analysis. After receiving a summary from the members, the lecturer modified it according to the questions raised, forming a draft for on-site collective lesson preparation. ③On-site collective lesson preparation: Regarding theoretical knowledge, the lecturer explained relevant knowledge, key links, and precautions. For the key links, the scientific predictions and reasonable mistakes during the operation procedures were emphasized and analyzed one by one. The operation demonstration was guided by clinical problems, based on the 6th edition of “Basic Nursing”, combined with clinical cases and preparation of standardized patients. Every operation procedure formed different but regular operation pathways, including quality requirements, pre-operation preparation, operation process, post-operation treatment, and effect evaluation. The scores were detailed for the operation assessment standards, thereby reducing the subjectivity of the invigilator. The group members expressed their own opinions, which were recorded by a designated person to form unified standards. ④Individual lesson preparation: Based on the collective lesson preparation earlier and the questions raised by group members, the lecturer conducted the second collective lesson preparation after 5 days to form a final standardized and homogenized operational teaching plan. The teaching course was recorded on video for trained nurses to watch repeatedly.

#### Skills training

The person in charge of the nursing training base arranged practice time based on the site conditions and published the available appointment time on the learning platform. Nursing staff could appoint practice time on the learning platform based on their work schedule. Members of the training group took turns to provide on-site guidance on operation practice to ensure the quality of practice. Nurses must log in to the platform and scan the QR code during practice. The backend system recorded everyone’s practice time and frequency. Each nurse must have at least two onsite practices per month to be eligible for the monthly draw. Failing to meet the daily practice standard would be deemed to have failed the exam. Table 2 shows the summary of the training program for the experimental group and the control group. The experimental group received skills training using a system that combines procedural pathways with information management, while the control group received traditional teaching mode. The training program lasted for one year, with monthly sessions of theoretical and skills training. The training content included patient admission and discharge care, hospital infection prevention and control, patient hygiene and safety care, patient balanced diet care, respiratory care, gastrointestinal care, specimen collection techniques, etc. The training mode consisted of online and offline teaching, collective and individual lesson preparation, operation demonstration and practice, and clinical supervision and feedback. The training assessment included theoretical score, operation score, nurse competency, patient satisfaction, and nursing-related adverse events.

**Table 2:** Summary of the training program for the experimental group and the control group

Group	Duration	Frequency	Content	Mode	Assessment
Experimental	One year	Monthly	Patient admission and discharge care, hospital infection prevention and control, patient hygiene and safety care, patient balanced diet care, respiratory care, gastrointestinal care, specimen collection techniques, etc.	Online and offline teaching, collective and individual lesson preparation, operation demonstration and practice, and clinical supervision and feedback.	Theoretical score, operation score, nurse competency, patient satisfaction, and nursing-related adverse events.
Control	One year	Monthly	Patient admission and discharge care, hospital infection prevention and control, patient hygiene and safety care, patient balanced diet care, respiratory care, gastrointestinal care, specimen collection techniques, etc.	Traditional teaching mode.	Theoretical score, operation score, nurse competency, patient satisfaction, and nursing-related adverse events.

### Effect evaluation

Nursing interns in the experimental and control groups underwent theoretical assessment, skills assessment, nurse competency assessment, patient satisfaction survey, and adverse nursing event statistics during the training period. The theoretical assessment, skills assessment, and patient satisfaction survey were conducted once a month, while the remaining assessments were performed at the end of the training.

The theoretical assessment was conducted in a closed-book format with a maximum score of 100, which indicated the trainees’ mastery of theoretical knowledge. The skills assessment was conducted by the supervising teacher. The nurses randomly selected basic and specialized operations for a total of 100 points, each with 50 points. The nursing operation assessment standards of the hospital were adopted, with higher scores indicating better clinical nursing performance of the trainees. The ICU nurse competency survey scale compiled by Qiao Anhua was used for nurse competency assessment, consisting of 58 items in 4 dimensions, including professional knowledge (14 items), professional skills (19 items), professional abilities (20 items), and psychological traits (5 items). The scale employed the Likert 5-point scoring, with a total score ranging from 0 to 232 points. A higher score indicated a higher level of competency, with a score of < 116 as fail, 116–173 as pass, and 174 as good. The Cronbach’s α coefficient of the scale was 0.93.

Adverse nursing events include: ①Poor treatment: including medication errors, blood transfusion errors, medical infection outbreaks, incorrect identification of surgical site, residual surgical instruments in the body, and infusion and transfusion reactions; ②Accidents: including falls, bed rail entrapment, wandering, burns, self-harm, suicide, fire incidents, theft, biting of thermometers, and improper restraint; ③Medical communication incidents: including disputes between medical staff and patients, physical attacks, fights, and violent behaviors; ④Incidents of poor diet and skin care: including accidental ingestion/choking, foreign body aspiration, hospital-acquired pressure ulcers, and healthcare-associated skin damage; ⑤Incidents of poor auxiliary diagnosis and patient transfer: including incorrect identification, specimen loss, sudden unexpected changes or accidents during examination or transportation; ⑥Incidents of poor catheter care: including tube dislodgement and patient self-removal; ⑦Occupational exposure: including needlestick injuries and cuts; ⑧Public facility incidents: including damage to hospital buildings, malfunction of ward facilities, deliberate destruction, and harmful substance leaks; ⑨Medical equipment incidents: including medical material failures, instrument malfunctions, and non-compliance with sterile requirements.

A patient satisfaction survey was conducted by the training group, with 40% of the total number of patients cared for by trained nurses being randomly selected for evaluation each month. The Newcastle Nursing Satisfaction Scale (NSNS) was adopted, with patients filling out questionnaires on their own. The evaluation content included nursing time, nurses’ ability, nurses’ visits to the ward, nurses’ affinity, nurses’ respect for patients, etc. Scores for each item were added up to calculate the total score, with higher scores indicating higher patient satisfaction.

### Statistical analysis

Statistical software SPSS 25.0 was used for data processing. Categorical data were expressed as frequency or rate and underwent the *χ*^*2*^ test. Measurement data obeying normal distribution were expressed as mean ± standard deviation (x ± s). An Independent sample t-test was used for inter-group comparison. Rank-sum test was used for ordinal data. *P* < 0.05 indicated a statistically significant difference.

## Results

### Comparison of skills and theoretical assessment results between two groups

The research results showed that after the nursing training and teaching, the monthly theoretical and clinical nursing operation scores of nursing interns in the experimental group were higher than those in the control group (*P* < 0.05), and the group differences were statistically significant. As the training time elapsed, the control group’s scores showed a slow upward trend, while the experimental group fluctuated up and down in a certain interval. The specific results are shown in Table 3.

**Table 3:** Comparison of Monthly Assessment Results of Nursing Theory and Skills in 2020 and 2021

Month	Assessment Scores ($\overline{x}\pm s$))							
	Control Group Theoretical Assessment	Experimental Group Theoretical Assessment	t value	*P* value	Control Group Skills Assessment	Experimental Group Skills Assessment	t value	*P* value
1	75.45$\pm$4.11	92.18$\pm$3.37	26.29	< 0.001	74.32$\pm$3.96	90.18$\pm$3.44	23.34	< 0.001
2	78.25$\pm$1.22	91.80$\pm$0.85	31.13	< 0.001	76.33$\pm$2.15	91.34$\pm$2.85	21.33	< 0.001
3	84.25$\pm$4.51	91.79$\pm$3.76	18.03	< 0.001	80.45$\pm$3.61	93.79$\pm$2.56	12.03	< 0.001
4	83.71$\pm$6.47	91.76$\pm$5.30	9.38	< 0.001	83.77$\pm$3.47	92.76$\pm$3.34	10.38	< 0.001
5	86.16$\pm$3.05	92.21$\pm$2.72	10.47	< 0.001	84.25$\pm$2.65	92.33$\pm$3.76	12.43	< 0.001
6	82.53$\pm$3.46	90.35$\pm$2.63	13.34	< 0.001	83.34$\pm$3.77	91.26$\pm$4.63	11.34	< 0.001
7	80.37$\pm$4.91	90.79$\pm$3.27	11.17	< 0.001	84.33$\pm$6.07	91.45$\pm$4.21	10.29	< 0.001
8	81.29$\pm$4.78	93.76$\pm$3.74	19.50	< 0.001	85.56$\pm$3.67	93.55$\pm$3.34	13.66	< 0.001
9	85.34$\pm$4.53	92.42$\pm$3.62	14.25	< 0.001	86.67$\pm$4.45	92.34$\pm$4.12	12.38	< 0.001
10	87.07$\pm$3.38	93.07$\pm$2.72	12.60	< 0.001	87.34$\pm$3.32	93.44$\pm$4.71	12.57	< 0.001
11	88.91$\pm$5.01	90.65$\pm$3.46	4.10	< 0.001	89.91$\pm$4.23	94.65$\pm$3.23	6.15	< 0.001
12	91.42$\pm$3.61	93.27$\pm$2.78	5.04	< 0.001	91.37$\pm$3.34	94.34$\pm$4.38	6.34	< 0.001

### Comparison of nurse competency between two groups

The assessment results of nurse competency showed that the total score, professional knowledge score, and professional skills score of nursing interns in the experimental group were 133.27 ± 9.95, 32.20 ± 3.00, and 44.60 ± 4.69, respectively, which were higher than those in the control group (122.88 ± 7.293, 28.38 ± 3.24, and 41.50 ± 2.76, respectively). *P* < 0.05, and the group differences were statistically significant. There was no statistically significant difference in professional abilities and psychological traits between the experimental group and the control group (*P* > 0.05). The specific results are shown in Table 4.

**Table 4:** Comparison of ICU nurse competency between two groups

Group	Experimental Group (*n* = 300)	Control Group (*n* = 267)	t value	*P* value
Total score	133.27 ± 9.95	122.88 ± 7.293	3.331	0.002
Professional knowledge	32.20 ± 3.00	28.38 ± 3.24	3.400	0.002
Professional skills	44.60 ± 4.69	41.50 ± 2.76	2.226	0.031
Professional abilities	43.13 ± 6.19	40.31 ± 5.26	1.370	1.818
Psychological traits	13.33 ± 1.54	12.69 ± 1.30	1.262	0.217

### Assessment of patient satisfaction for two groups

A survey was conducted on patient satisfaction with nursing. The evaluation content included nursing time, nurses’ ability, nurses’ visits to the ward, nurses’ affinity, nurses’ respect for patients, etc. The comparison results of total score showed that there were statistically significant differences in monthly scores between the experimental group and the control group (*P* < 0.05), and the scores of the experimental group were higher than those of the control group. The results are shown in Table 5.

**Table 5:** Comparison of monthly patient satisfaction between two groups

Month	Total Satisfaction ($\overline{x}\pm s$)		t value	*P* value
	Control Group	Experimental Group		
1	89.45$\pm$6.75	92.94$\pm$5.53	3.25	0.0015
2	90.09$\pm$4.64	92.32$\pm$3.82	3.04	0.0029
3	91.88$\pm$6.31	95.86$\pm$5.26	3.93	0.00014
4	91.09$\pm$7.33	96.39$\pm$6.15	4.538	< 0.001
5	92.71$\pm$3.03	98.93$\pm$2.01	13.99	< 0.001
6	92.48$\pm$4.31	98.31$\pm$3.04	8.98	< 0.001
7	94.22$\pm$2.67	99.33$\pm$1.90	12.96	< 0.001
8	93.63$\pm$3.07	98.73$\pm$2.50	10.62	< 0.001
9	94.47$\pm$4.18	98.45$\pm$3.07	6.33	< 0.001
10	93.84$\pm$3.19	98.97$\pm$2.08	11.10	< 0.001
11	95.71$\pm$2.89	99.55$\pm$1.04	10.32	< 0.001
12	96.75$\pm$3.02	99.36$\pm$1.47	6.41	< 0.001

### Comparison of adverse nursing events between two groups

**Fig. 1:**
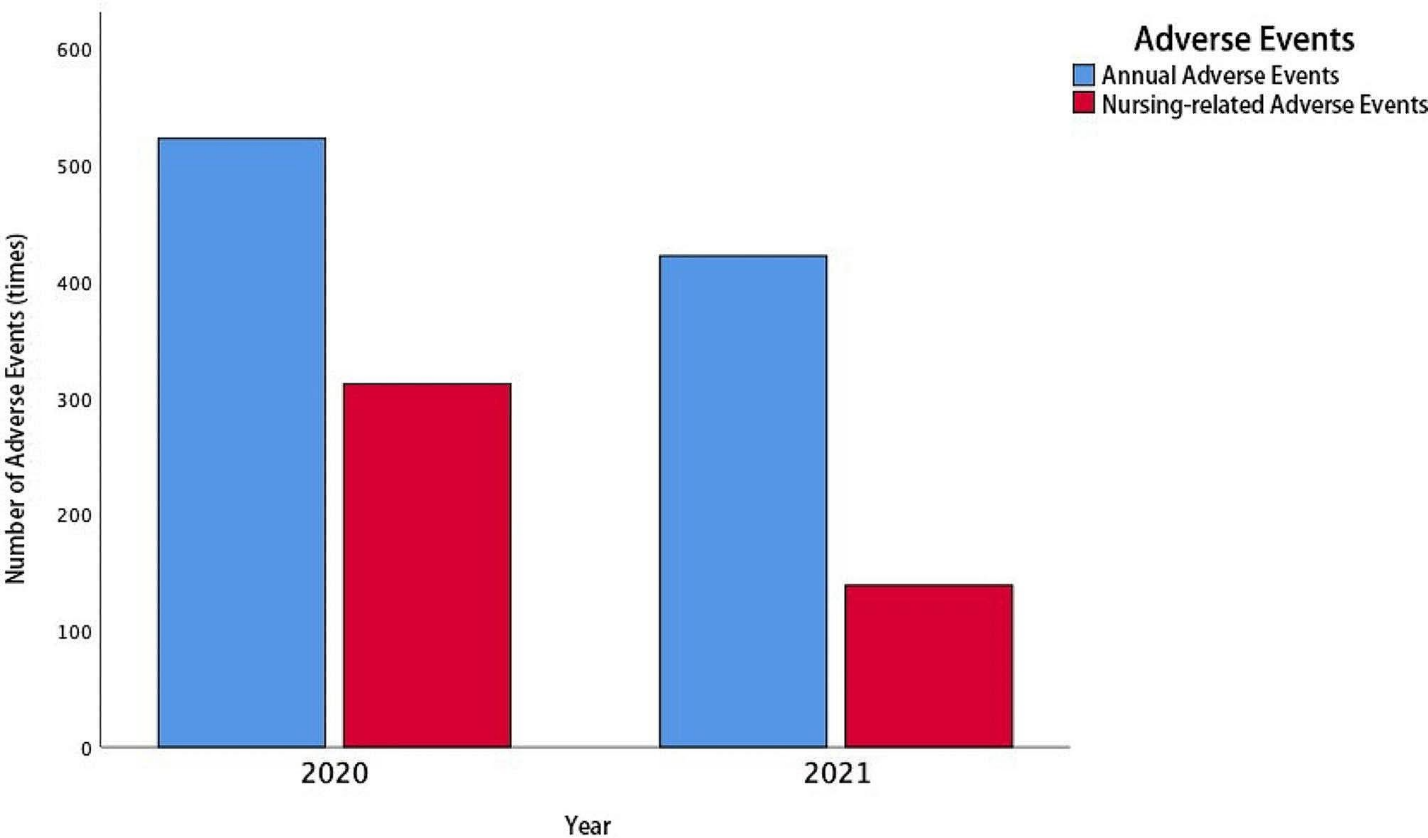
Incidence of Adverse Events in 2020 and 2021

The incidence of adverse events in 2020 and 2021 is shown in Fig. 1. As can be seen, compared with 2020, the total number of adverse events and nursing-related adverse events in 2021 both decreased. Upon further comparison of nursing-related adverse event incidence in 2020 (control group) and 2021 (experimental group), it was found that the incidence of nursing-related adverse events in the two groups was statistically significant (*χ*^*2*^ = 68.824, *P* < 0.001), as shown in Table 6.

**Table 6:** Comparison of adverse events caused by improper nursing operations in 2020 and 2021

Year	Nursing-related adverse events *n* (%)	${\chi }^{2}$ value	*P* value
2020	312(59.66)	68.824	*P* < 0.001
2021	139(32.94)		

### Discussion

Nursing is inherently a practice-oriented discipline, with clinical nursing skills constituting an essential aspect of vocational competencies [9, 10]. This study introduces an innovative approach by integrating procedural pathways with information management within a nursing skills operation group. The objective is to enhance the safety and standardization of nursing operations in clinical settings, thereby improving the overall quality of nursing and safeguarding patient safety. The empirical evidence suggests that nurses in the experimental group, who were exposed to this combined strategy, outperformed those in the control group across various metrics such as assessment scores, competency, and patient satisfaction, with statistical significance (*P* < 0.05). This improvement is attributed to the blended learning approach that transcends the barriers of time and space, leveraging both online and offline resources for teaching, and fostering a cohesive synergy among various educational elements and dimensions. The platform facilitates continuous online learning and interactive teacher-student engagement, thus enhancing the quality and efficiency of instruction. The specific advantages of this system are:

By promoting resource sharing, the system benefits both instructors and students. Collective lesson preparation is a critical step in the teaching process, with proper planning serving as the foundation for successful task completion [1, 11]. It creates a collaborative environment where skills operation group members, drawing from their theoretical knowledge and clinical experience, can engage in dynamic exchanges, intellectual discussions, and meticulous deliberation, leading to an enriching interaction of knowledge and practice. This collective process fosters an educational culture where the nursing skills group leads and the broader nursing staff actively participates, ensuring access to high-quality education for all hospital nurses.

The system refines training strategies and fortifies basic nursing practices. Training should align with clinical demands and focus on job competency [12]. It has been observed that the challenge for nurses is not the lack of understanding of nursing procedures, but difficulty in retention [13]. By converting these procedures into procedural pathways and incorporating scientific error prevention and management, the system bridges theory with practice. Through video-based error analysis and on-site clinical supervision, the training process becomes more engaging and practically oriented, thereby enhancing the quality of clinical nursing and embracing a patient-centered service ethos.

The system is instrumental in managing nursing quality control and elevating nursing standards. With patient safety as a global healthcare priority, minimizing medical errors or adverse events is paramount [14]. Adverse nursing events are often linked to operational behaviors [15, 16], and traditional training methods tend to overlook the development of problem-solving skills in real-world settings [17]. This system’s synchronous delivery of theory and practical procedures equips nurses with a thorough understanding and mastery of operational steps, which has been evidenced by a notable reduction in adverse events (refer to Table 2) and, consequently, improved nursing quality. Moreover, the inclusion of standardized patient assessments bolsters clinical reasoning skills, moving away from rote learning of nursing techniques [18, 19].

Lastly, the system circumvents negative factors and aids in fostering a magnet hospital environment. Conventional nursing training, which is heavily reliant on live demonstrations and hands-on teaching, can be time-consuming for instructors and nurses alike, often leading to reluctance and resistance [20]. This study demonstrates that the use of video-based learning modules, which nurses can access at their convenience, not only respects their time but also encourages self-directed learning and enhances training outcomes [21, 22]. This approach exemplifies a commitment to humanistic care for nursing staff and mitigates any resentment stemming from the encroachment of training on their personal time. In the current digital age, where hospitals are rapidly advancing their clinical information systems, this study’s learning platform stands out by offering a flexible, adaptable, and efficient training model that aligns with the digital transformation in healthcare education [23].Our findings are consistent with those of previous studies that have reported the positive effects of procedural pathways and information management on nursing skills training and clinical nursing quality [24–26]. However, our study also contributes to the literature by providing a comprehensive and systematic evaluation of the application effect of this strategy, using multiple indicators and methods, such as theoretical and skills assessment, nurse competency assessment, patient satisfaction survey, and adverse nursing events statistics. Moreover, our study is one of the first to explore how this strategy can promote the construction of a magnet hospital, which is a key factor for attracting and retaining qualified nurses and improving patient outcomes [27–29].

Despite this, this study has some limitations. First, the small sample size resulted in low test efficiency, leading to differences in some items showing no statistical significance between the experimental group and the control group. Second, as it was difficult to include samples, the control group was based on previous data and did not follow the principle of random grouping. Further improvement should be made in these two aspects for in-depth exploration.

### Conclusion

In summary, nursing operation skills are a focal point of nurse training and a crucial constituent of nurses’ core competency [30], which is closely linked to clinical quality and safety [31]. Within this system, rigid nursing skills are presented through a humanized training system, which combines theoretical knowledge with clinical practice to gradually form a procedural pathway that integrates standardization, learning, training, assessment, analysis, and feedback. The introduction of informatization has rendered the training process more targeted, strategic, and organized. The convergence of the two can effectively improve the training effect and nursing quality and has been acknowledged by the nursing staff.The application of the results of this study can provide a new perspective and method for nursing operation skills training, which can be used by other hospitals or nursing institutions to improve their training quality and effectiveness. This study also contributes to the literature by providing a comprehensive and systematic evaluation of the application effect of procedural pathways combined with information management, using multiple indicators and methods. Moreover, this study is one of the first to explore how this strategy can promote the construction of a magnet hospital, which is a key factor for attracting and retaining qualified nurses and improving patient outcomes.

## Data Availability

All data generated or analyzed during this study are included in this published article.
